# Patient Perceptions of Medicare Annual Wellness Visits: Findings From a Pilot Survey

**DOI:** 10.1111/jgs.70564

**Published:** 2026-06-23

**Authors:** Amy Cunningham, Lauren Hersh, Brooke Salzman, Kristine Swartz, Michael Danielewicz, Rebecca Berger, Elizabeth Clark, Payton McCready, Tushar Kumar, Stephanie Michael, Susan Mockus Parks

**Affiliations:** ^1^ Department of Family and Community Medicine Sidney Kimmel Medical College at Thomas Jefferson University Philadelphia Pennsylvania USA; ^2^ Sidney Kimmel Medical College at Thomas Jefferson University Philadelphia Pennsylvania USA

**Keywords:** Medicare Annual Wellness Visit, patient satisfaction, survey

## 1 Introduction

Recognizing the need for better preventive care, Medicare introduced the Annual Wellness Visit (AWV) in 2011 via the Affordable Care Act in an effort to encourage the adoption of preventive health services by older adults. Through the AWV, clinicians develop personalized prevention plans which mitigate disease and disability based on a beneficiary's health and risk factors. The AWV includes a health risk assessment, family and medical history, current healthcare providers and medications, cognitive impairment and depression screenings, and preventive services counseling and referrals [1]. AWVs have yielded multiple benefits, including increased use of preventive services [2, 3]; however, AWV completion rates are suboptimal in many health systems [4]. Understanding patient satisfaction with AWVs and ways to improve the patient experience may improve completion rates. Despite this need, research on patient satisfaction with AWVs is limited [5, 6, 7, 8], and most studies assess patient satisfaction with pharmacist‐led AWVs [6, 7, 8], rather than physician or nurse‐led AWVs.

Jefferson Health, a large health system in eastern Pennsylvania and Southern New Jersey, is a participant in the John A. Hartford Foundation and Institute for Healthcare Improvement's Age‐Friendly Health Systems Initiative [9]. Jefferson's Center for Healthy Aging (CHA) has embarked on an Age‐Friendly redesign of its AWVs. The purpose of this study was to obtain baseline data on Medicare patients' experience and satisfaction with AWVs in a Jefferson Health geriatrics practice.

## 2 Methods

This cross‐sectional survey study took place at Jefferson Health's CHA, a geriatrics practice with approximately 2000 patients located in downtown Philadelphia. Eligible survey participants were CHA patients. An eight‐item survey was developed based on a comprehensive literature review and consultation with practice clinicians. Survey items assessed patient familiarity with the Medicare AWV, whether the patient had completed an AWV, the importance of the AWV provider being a physician, and their satisfaction with the length of the visit, the information received, and the issues discussed during the visit. Patients were also asked if they made changes or followed suggestions based on the AWV, and their overall satisfaction with the AWV.

The survey was distributed to eligible patients via the Epic MyChart messaging system in July 2025. Patients were electronically sent the survey one time. Descriptive analyses were performed on the survey data using IBM SPSS. The study was approved as exempt by Thomas Jefferson University's Institutional Review Board.

## 3 Results

Four hundred seventy‐eight patients (24%) completed the survey. Seventy‐three percent were familiar with Medicare AWVs, and 64% completed one. Of those who had completed an AWV (*n* = 301), 63% said it was important or very important that the provider be a physician. Patient experience and satisfaction data for patients who completed a Medicare AWV are shown in Figure 1. The majority of participants were satisfied/very satisfied with visit length (70%), information provided (73%), and issues discussed (76%). Sixty percent reported making changes or following suggestions based on discussion with the AWV provider. Overall, 74% were satisfied/very satisfied with the AWV.

**FIGURE 1:**
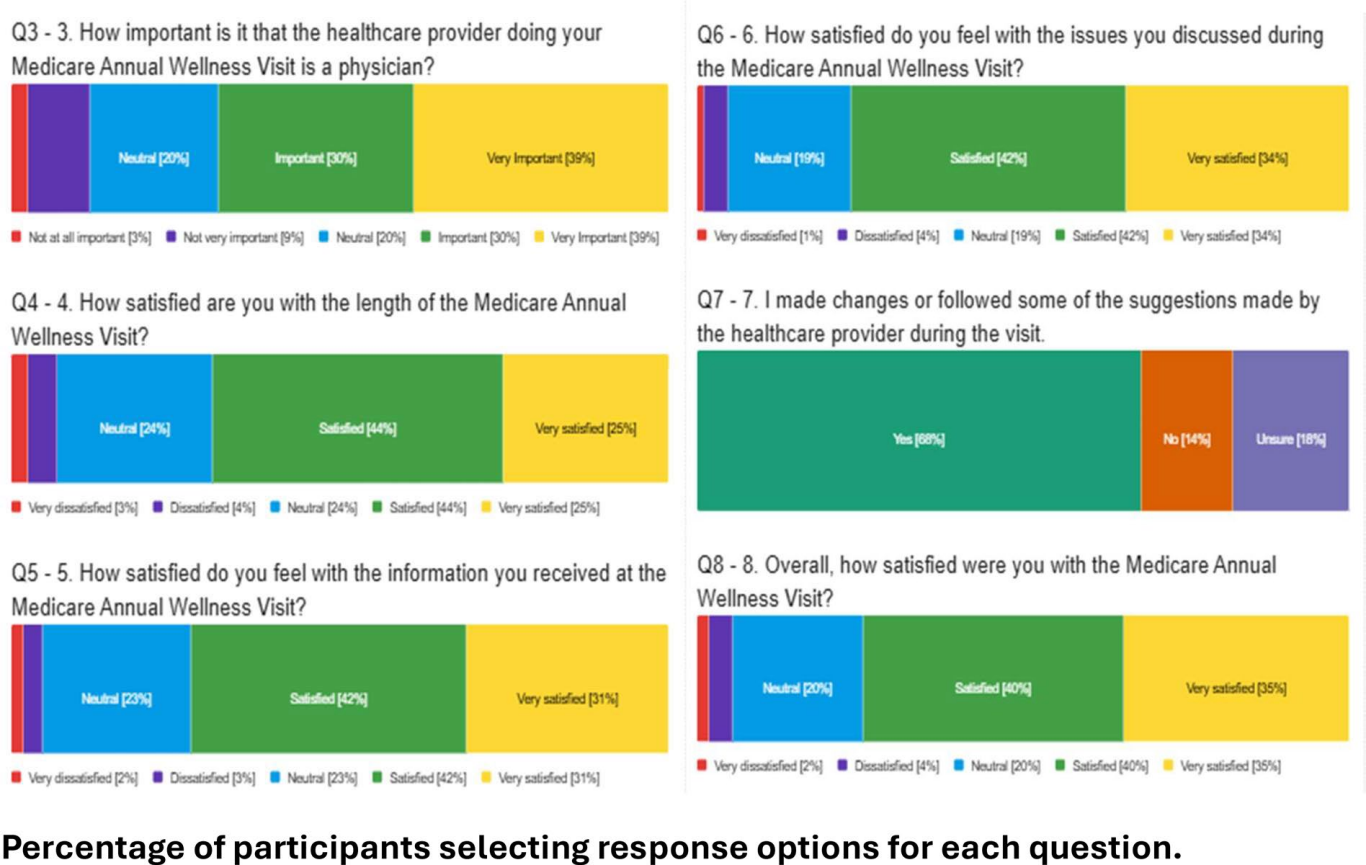
AVW patient experience and satisfaction data (*N =* 301). This figure summarizes the responses to six questions from the Medicare Annual Wellness Visit Survey. For Question 3. responses are reported as percentage of respondents selecting “Not at all important,” “Not very important,” “Neutral,” “Important,” or “Very Important.” For Questions 4, 5, 6, and 8, responses are reported as percentages of respondents selecting “Very dissatisfied,” “Dissatisfied,” “Neutral,” “Satisfied,” or “Very Satisfied.” For Question 7, responses are reported as the percentage of respondents selecting “Yes,” “No,” or “Unsure.”

## 4 Discussion

In this survey of patients in a geriatrics practice, over 70% of respondents were familiar with Medicare AWVs. The response rate showed that administration of a survey via MyChart was feasible. Baseline satisfaction with AWVs was high, including the visit length and issues discussed. Notably, 60% reported making changes or following suggestions based on the AWV, reinforcing the role of the AWV as an important preventive opportunity. Our survey did not capture what changes patients made; this would be an important area for future research to fully capture the impact of AWVs.

Also striking was the high percentage of respondents who felt it was important or very important that their AWV provider be a physician. The authors questioned whether patients interpreted this question to mean their own physician or simply a physician in general, highlighting an area for further exploration. Efforts to increase AWV completion rates have involved authorizing non‐physician practitioners and other medical professionals to conduct AWVs, with positive results [10]. Studies on pharmacist‐led AWVs report high patient satisfaction and found that most patients did not specifically prefer to see their primary care physician for their AWV [6, 7, 8].

This study has several limitations. First, the survey response rate may have contributed to response bias. Second, the survey was designed to be a brief, anonymous survey to encourage completion; this also meant that we did not collect demographic data on respondents. Finally, the survey was completed by patients at a single geriatrics practice in Philadelphia, which may limit its generalizability. A follow up survey will be conducted in Summer 2026 to assess patient attitudes and satisfaction after the implementation of Age‐Friendly Medicare AWVs at this practice.

## Author Contributions

Study concept and design: A.C., B.S., K.S., P.M., M.D., and S.M.P. Acquisition of subjects and/or data: A.C., B.S., S.M.P. Analysis and interpretation of data: A.C., B.S., K.S., P.M., M.D., S.M.P., R.B., E.C., T.K., and S.M. Preparation of manuscript: A.C., B.S., K.S., P.M., M.D., S.M.P., R.B., E.C., T.K., and S.M.

## Funding

The authors have nothing to report.

## Disclosure

The authors have nothing to report.

## Conflicts of Interest

The authors declare no conflicts of interest.
